# Involving young people in BRIGHTLIGHT from study inception to secondary data analysis: insights from 10 years of user involvement

**DOI:** 10.1186/s40900-018-0135-x

**Published:** 2018-12-27

**Authors:** Rachel M. Taylor, Jeremy S. Whelan, Faith Gibson, Sue Morgan, Lorna A. Fern, on behalf of the BRIGHTLIGHT Team, Natasha Aslam, Julie Barber, Richard Feltbower, Zuwena Fox, Louise Hooker, Sarah Lea, Martin Lerner, Ana Martins, Tony Moran, Steve Morris, Catherine O’Hara, Nishma Patel, Susie Pearce, Rosalind Raine, Anita Solanki, Dan Stark, Cecilia Vindrola, Young Advisory Panel (YAP), Zeena Beale, Ciaran Fenton, Emily Freemantle, Laura Haddard, Steph Hammersley, Amy Lang, Joshua Lerner, Tanya Loughlin, Maria Onasanya, Steph Still, Poppy Richards, Amy Riley, Freya Voss, J. J. Wheeler, Antonia Young, Core Consumer Group (CCG) and National Cancer Research Institute Teenage and Young Adult Clinical Studies Group (NCRI TYA CSG)

**Affiliations:** 1 Cancer Division, University College London Hospitals NHS Foundation Trust, London, UK; 2 School of Health Sciences, Faculty of Health and Medical Sciences, University of Surrey, Guildford, Surrey GU2 7XH UK; 3 Centre for Outcomes and Experience Research in Children’s Health, Illness and Disability (ORCHID), Great Ormond Street Hospital for Children NHS Foundation Trust, London, UK; 4 Teenage Cancer Trust Unit, Leeds Teaching Hospitals NHS Foundation Trust, Leeds, UK

**Keywords:** Adolescents, Young adults, Teenagers, Cancer, Recruitment, Retention, Dissemination, BRIGHTLIGHT, Patient and public involvement, Consumers

## Abstract

**Plain English summary:**

Young people with cancer are often described as ‘hard to reach’, ‘difficult to engage’ and/or ‘vulnerable’. Consequently, they are often over looked for patient and public involvement activities. We set out to involve young people with cancer to work as co-researchers in the design of the largest ever study of young people with cancer, called BRIGHTLIGHT. In the 10 years since the BRIGHTLIGHT feasibility work began we have involved more than 1200 young people as co-researchers, collaborators, consultants and dissemination partners. We chronicle the key points of this 10-year journey, sharing our success, describing our challenges and the solutions we put in place; sharing also what worked and did not work. Here we share some of these experiences of involving young people in this research and offer some practical advice for those looking to do the same.

**Abstract:**

**Background**

Young people with cancer, broadly those aged 13–24 years at diagnosis, warrant special attention; physiological and psychological growth creates complex psychosocial needs which neither adult nor child systems are suitably designed to deal with. Resulting from these needs, they are often described as ‘vulnerable’, ‘hard to reach’ and ‘difficult to engage’, and consequently are often over looked for patient and public involvement/engagement (PPIE) roles. In our study ‘BRIGHTLIGHT’, we set out to evaluate whether specialist care for young people adds value, ensuring young people were central to our PPIE activities. We believe that BRIGHTLIGHT is unique as a very large study of young people with cancer which has successfully overcome the challenges of including young people in the research process so we are confident that they have influenced every aspect of study design, conduct and dissemination.

**Methods**

We chronicle a period of 10 years, over which we describe our approach and our methods to involving young people in PPIE activities in BRIGHTLIGHT. We describe the feasibility work, study set up, conduct and dissemination of our findings, and weave through our story of PPIE to illustrate its benefits. Through the narration of our experience we highlight significant points that both influenced and changed our direction of travel. We reflect on our experiences and offer some practical advice for those looking to do the same.

**Results**

In the 10 years since the BRIGHTLIGHT feasibility work began we have involved more than 1200 young people. Their contributions have been isolated and mapped over a 10-year period. We begin at an early step of identifying what research questions to prioritize, we then plot PPIE activities for one of these research priorities, place of care, which evolved into BRIGHTLIGHT. We document steps along the way to evidence the impact of this involvement.

**Conclusions**

Young people can make a valuable contribution to healthcare research given adequate support from the research team. Although some challenges exist, we propose that the benefits to young people, researchers and the study considerably outweigh these challenges and PPIE with young people should be integrated in all similar research studies.

## Introduction

Patient and public involvement and engagement (PPIE) is central to healthcare delivery and since the National Institute for Health Research (NIHR) launched INVOLVE in 1996, involving patients has also become central to health services research in the United Kingdom (UK) [1]. Much has been written about the challenges, barriers and facilitators of involving patients in research [2–4] and work evaluating PPIE has shown it to be a positive experience for patients and researchers. It can also have a positive influence on the research itself, such as increasing recruitment [5–7].

One of the challenges of PPIE for researchers is involving populations typically described as ‘hard to reach’, ‘hard to engage’ or ‘vulnerable’. It is however important that these populations are included to ensure research is relevant and accessible to all patients so that subsequent results are more reflective of the total population and not just selected groups [8]. In recognition of the need to include underrepresented populations researchers are striving to be more inclusive, for example, work is now reported on involving children [6], patients with mental illness [9] and disadvantaged pregnant women [10].

The aim of this paper is to review our own progress and reflect on our 10-year experience of user involvement with teenagers and young adults (TYA) with cancer, one of the populations often described as hard to reach [11]. We present an evolving picture of engagement from the outset, from developing our research question to underpin an NIHR programme grant, through to the dissemination of findings. We describe how TYA have been actively involved in every stage of the research process and how this has impacted on the study and those involved in PPIE activities. We conclude by drawing on our experience to provide guidance to other researchers, which we describe as ‘the 7Ps of PPIE’.

This article is an overview of our experience of involving young people with cancer in research. One of our early challenges was the lack of published guidance or examples of PPIE in research, particularly when working with young people. Therefore, from the outset we committed to publishing and generating evidence to support others developing PPIE. Accordingly, this paper does not present detailed methodology of PPIE activities and readers are referred to existing publications [5, 12–16]. We hope these reflections will inspire other researchers to commit to PPIE and give insights to further enhance its contribution in existing practices in order to progress the ‘science’ of user involvement and engagement, particularly with underserved groups.

## Involving young people with cancer in research, how we began

The chronology of our study called BRIGHTLIGHT, and the associated patient involvement is presented in Table 1. The prelude to establishing a TYA user group was the publication of the National Institute for Health and Care Excellence (NICE) guidance in 2005 on the provision of cancer care for children and young people aged 0–24 years at diagnosis [17]. This was in response to the increased awareness of poorer outcomes for TYA [18–20] and directed that young people aged 16–18 *must* be referred to a specialist TYA cancer unit and those aged 19–24 should be offered a choice of where to have their treatment but have ‘unhindered access’ to age appropriate services [17]. The guidance for children was based on robust evidence, a consequence of a highly co-ordinated approach to children’s cancer care and research, however the evidence on which to base guidance for TYA cancer care was insubstantial and recommendations were based on best available evidence and expert opinion (Whelan personal communication).

**Table 1:** Chronology of BRIGHTLIGHT patient involvement

Year	Activity
2008	Core Consumer Group (CCG) present at Find Your Sense of Tumour (FYSOT)
2009	Essence of Care study begins
	CCG conduct peer-to-peer interviews
	CCG conduct data analysis
	CCG member assists with facilitating professional workshop
2010	CCG conduct second consultation at FYSOT
	CCG present at Plenary Session National Cancer Research Institute (NCRI) Conference
	CCG present at INVOLVE conference
2011	CCG present Essence of Care results at FYSOT
	Workshop to present Essence of Care results back to CCG and study participants
	Workshop to create study name: BRIGHTLIGHT
2012	BRIGHTLIGHT logo launched
	Focus groups to develop the BRIGHTLIGHT survey Young Advisory Panel (YAP) workshop to develop the website
2013	YAP workshop on access to research
	YAP instigate launch of Twitter
2014	YAP member interviewed by BBC5 Live Radio
	YAP members create video of information sheets
	YAP workshop on study retention
	BRIGHTLIGHT public and patient involvement and engagement (PPIE) featured in the NCRI newsletter
	YAP present at FYSOT
2015	BRIGHTLIGHT featured in INVOLVE newsletter
	YAP workshops - generating hypotheses, and body image and sexuality
	YAP launch new website
2016	YAP workshop on online information needs
	YAP member presents at NCRI Conference Schools Session
2017	Begin production of dissemination play, *There is a Light*
	Opening night of *There is a Light* at the S!CK festival
	YAP co-chair the joint TYAC-BRIGHTLIGHT conference
	YAP workshop on sexuality

*CCG* Core Consumer Group, *FYSOT* Find Your Sense of Tumour, *NCIN* National Cancer Intelligence Network, *NCRI* National Cancer Research Institute, *PPIE* public and patient involvement and engagement, *RCN* Royal College of Nursing, *YAP* Young Advisory Panel

In response to this lack of research evidence, in 2005 the National Cancer Research Institute (NCRI) created a TYA-specific Clinical Studies Group (CSG) tasked with generating research specifically for young people, broadly those aged 13–24 at diagnosis and the Group was supported by an independently funded researcher (Lorna Fern). Specifically within its remit, and in response to the limited evidence base for TYA in the NICE guidance, ‘*To generate research into the optimal provision of health care for patients in that age group and in particular to provide the evidence base for the present and future NICE Improving Outcomes Guidance for children and young people with cancer’.*

The NCRI CSGs have PPIE embedded in their structure and two consumer representatives are full members of each group. Recognising the diversity of life stage commitments, cancer types and age range, the inaugural Chair of the TYA CSG (Jeremy Whelan) successfully argued that the TYA CSG might be better assisted by an alternative model of involvement. After a series of workshops and consultation with young people to consider different approaches to involvement, five young people, who named themselves the TYA CSG Core Consumer Group (CCG), were appointed for a fixed term to provide consumer representation for the TYA CSG. The CCG were mentored by two TYA nurses and the work was led by the funded researcher (Lorna Fern). The mentoring role of the nurses had two functions, firstly to support the young people with their understanding of the research processes and complex terminology, guiding young people through protocols and assisting with understanding research results. The second function was assisting the researcher, who, as a non-clinician had not previous contact with young people with cancer at that time. The nurses would review the documents prepared by the researcher for terminology and sense prior to them being sent to the CCG. In the beginning the mentors would also relay any anxieties or problems back to the research team on behalf of the CCG; however, with time this function was not required as the CCG quickly became confident to find their own voice and discuss any issues directly with the research team.

One of the first tasks assigned to the CCG was to seek consultation from a wider group of young people and prioritise areas of research identified by the professionals on the TYA CSG. With support from the two TYA nurses and the researcher the CCG designed an information leaflet, a presentation and key questions to ask young people attending a Teenage Cancer Trust’s annual patient conference attended by over 200 young people with cancer, ‘Find Your Sense of Tumour’ (FYSOT), https://jtvcancersupport.com/2008/10/fysot09-ncri/: Which of the research projects [presented earlier by the CCG] did you think was the most important?’ Listed in their order of priority they were:

1. Delays in cancer diagnosis.
2. Survivorship and late effects.
3. Place of care.
4. Access to clinical trials.
5. Information collection.

What follows, and forms the majority of our paper, is the detail of PPIE activities that underpinned priority 3, ‘place of care’; a study that evolved and was renamed as ‘essence of care’ then ‘BRIGHTLIGHT’ [14]. Place of care described a proposed study to examine whether young people benefit from receiving treatment in a specialist age-appropriate environment and included the overall philosophy of how care is delivered.

## Our first study: Essence of care

The NICE improving outcomes guidance document [17] set out how young people with cancer should be cared for. Four key questions arose for which methodology required to address them was not initially apparent (Table 2). The Health Services Research subgroup of the TYA CSG received funding from Teenage Cancer Trust to conduct a series of feasibility and exploratory studies to: 1) understand TYA cancer services; 2) address the methodological issues related to evaluating complex service configuration; and 3) develop the management and organisational structure of a programme of research (which in time became BRIGHTLIGHT). Collectively these series of projects formed the ‘Essence of Care’ study. It was intended from the outset to work with young people as co-researchers, recognising that a study about young people with cancer would be more relevant if designed by young people themselves.

**Table 2:** Unanswered questions supporting TYA cancer care

1. What is age appropriate specialist care? 2. Which are the most important elements of a TYA service? 3. What outcomes are improved by this service? 4. How much does this cost the young people, their families and the NHS?	

The CCG were involved in three core aspects of the Essence of Care project:

1. Identifying priorities for an age-appropriate environment.
2. Describing young peoples’ experience of care.
3. Identifying the primary outcome measure for a study which would measure whether age appropriate care adds value.

This involved co-designing workshops, co-facilitation of workshops, undertaking data collection with young people, and participating in dissemination. This work has been described in detail elsewhere [12, 13], and summarised here as context for the rest of this paper.

### Identifying priorities for an age-appropriate environment

The aim was to identify key components of a specialist TYA cancer unit from the perspective of young people and healthcare professionals. Members of the CCG collaborated with researchers to lead a workshop with young people who were either undergoing or had completed treatment. Participants were given cards containing key features of an inpatient ward and those proposed for ‘age-appropriate’ care as described in academic literature [21]. Through consensus they were asked to prioritise importance of individual features on a pyramid. The CCG felt that more young people should be consulted and so further consultation was sought from the participants of FYSOT in 2010. The research team conducted the same exercise with healthcare professionals and a comparison of the results showed young people prioritised factors that would help them through treatment, such as a dedicated unit and peer support. In contrast, healthcare professionals cited priorities such as clinical expertise [12]. This discrepancy in priorities emphasised the need for the proposed programme of research to be informed by young people and illustrated how a detailed exploration of the environment of TYA cancer care needed to be through both the lens of those receiving care as well as those delivering it.

### Describing young peoples’ experience of care

A literature review identified that there was a dearth of information about young peoples’ experience of care or place of care [22]; their expectations of healthcare services; information needs and perceptions of change in life situation. The CCG had received training in research methods^1^ and with additional support from the research team became co-researchers in a study focussing on these missing experiences. They designed an interview schedule and conducted peer-to-peer interviews with 11 young people. The interviews concluded with participants describing one of their key experiences as a fictional newspaper headline. To add detail and clarify points raised in the interviews, the headlines were used as guides for the focus of group discussion. This highlighted how important the young people regarded all staff members they had contact with from consultants to cleaners. The CCG worked with the research team to analyse the data and named the emergent themes [13].

These key themes (Table 3) were then presented by the CCG to young people attending FYSOT in 2011 to confirm they were appropriate and feedback was sought on any missing domains https://jtvcancersupport.com/2011/03/fysot-11-ncri-research-for-you/. The information from the peer interviews also helped inform the content of the BRIGHTLIGHT Survey to be used later in the longitudinal cohort study [23].

**Table 3:** Fictional newspaper headlines and the resulting theme

Newspaper headline	Resulting theme
Cancer diagnosis made me grow up	Life changing impact of diagnosis
I’m more than my cancer	Provision of information
If I’d had known… I would have travelled there	Place of care
Cancer nurse tells mum to get out)	Role of health professionals
It’ll finish one day, treatment’s not forever	Coping
Rehab[ilitation] buddies for cancer survivors	Peer support
Counselling for patients to cope	Psychological support
The tumour’s out but what now	Life after cancer

**Fig. 1:**
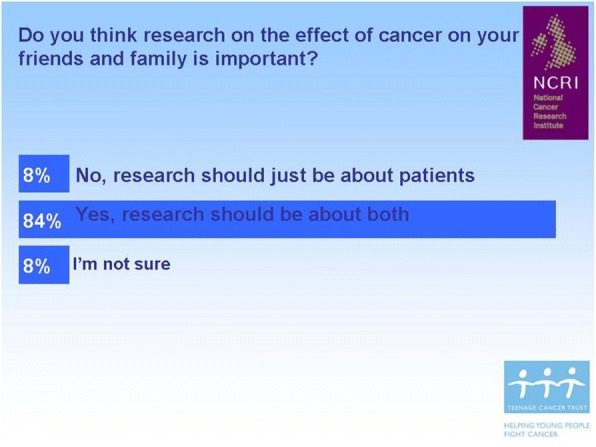
The majority of young people feel cancer research should also include families and friends

During discussions arising in the workshop, young people felt that cancer research should also focus on the impact of cancer on their family and friends. The CCG suggested asking a wider audience and again we utilised FYSOT 2010 and asked over 200 young people whether they thought cancer research for young people should also include the impact of cancer on others (Fig. 1). In recognition of this result, we included a questionnaire to be answered by caregivers alongside the BRIGHTLIGHT survey at the first time of data collection, administered to the person young people nominated as their main caregiver.

### Identifying the primary outcome measure

**Fig. 2:**
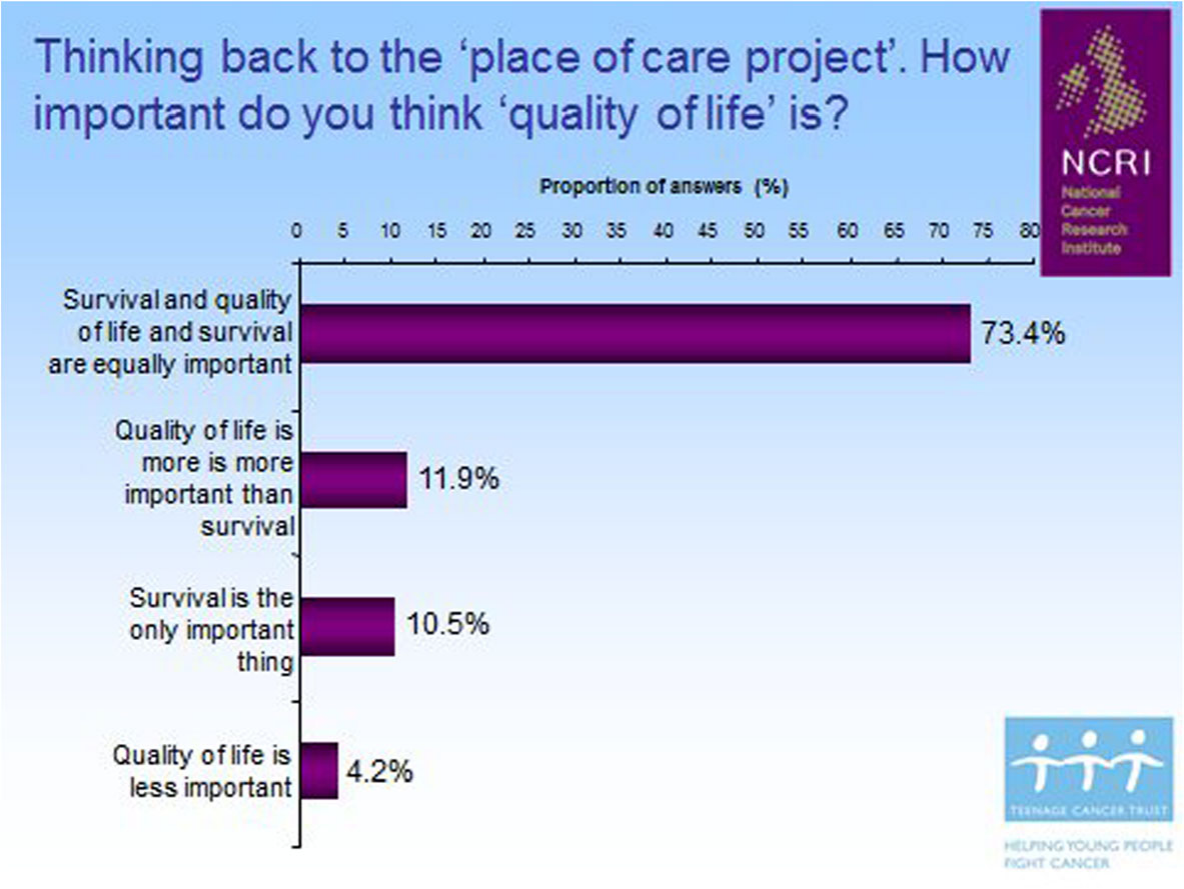
Young people feel that quality of life is as important as survival

Identifying the appropriate primary endpoint that would measure whether age-appropriate care adds value was a central goal of the feasibility work. The literature review and the cancer experiences workshop consistently identified quality of life as a potential outcome, which might be influenced by access to specialist care [12, 13]. After the CCG gave a short presentation at FYSOT to introduce quality of life as a measurable outcome, young people were asked to decide how important survival was in relation to quality of life. Approximately 75% of young people felt that quality of life and survival were equally important (Fig. 2).

## From essence of care to BRIGHTLIGHT

The Essence of Care results informed the grant submission: ‘Do specialist cancer services for TYA add value?’, which was successfully funded in December 2011 by the National Institute for Health Research (NIHR) as a Programme Grant for Applied Research (RP-PG-1209-10013), with a member of the CCG named and funded as a co-applicant on the grant. We were keen to continue to have young people at the heart of the study, however we felt a new and more responsive model of involvement was required, respecting the life stage commitments of young people and time commitment required to work alongside the team as co-researchers over a long period of time.

The CCG suggested that ‘Essence of Care’ might be improved on as a name for the study, a viewpoint verified by the participants of FYSOT 2011 [14]. Our first priority was therefore to establish a patient user group and rename the study.

Naming research studies is usually researcher driven and hence does not always appropriately reflect the study or its target population. For example, Angina With Extremely Serious Operative Mortality Evaluation known as AWESOME [24] does not necessarily reflect the experience of patients with angina. Study naming is valuable, it assists branding and ease of recognition, for healthcare professionals it can make it easier to refer to a study and can give meaning to participants if they identify with the name. We aimed to involve young people in naming and branding our newly funded research programme. Using methods derived from market research young people successfully created the name ‘BRIGHTLIGHT’ (‘Light at the end of the tunnel, leading the way for other young people’) and provided the mood board that guided logo design [14].

## What is BRIGHTLIGHT?

BRIGHTLIGHT is an evaluation of cancer services in England for young people aged 13–24 years at diagnosis designed to answer the outstanding questions from the NICE guidance [17] (Table 2). To determine whether specialist cancer services added value a series of six research projects ran concurrently to provide a comprehensive evaluation of the environment of care, people delivering care, young people and carers experience of care and finally the cost of TYA cancer care (Table 4; http://www.brightlightstudy.com/). While the guidance applied to young people aged 16–24 years, the inclusion criteria for BRIGHTLIGHT was lowered to 13 years to reflect the variation in service delivery across England. The first activity was the branding workshop in December 2011, recruitment of young people began in 2012 and, due to recruitment challenges, the core Cohort study [11, 25], was not completed until 2018.

**Table 4:** A summary of the BRIGHTLIGHT programme of research

Aspect of evaluation	Study design
Workstream 1: What is specialist care?	
Identify the competence of healthcare professionals delivering care to TYA	International e-Delphi study [31]
Describe the culture of TYA cancer care and define ‘age-appropriate care’	Multi-case study across 4 networks of care (involving 24 NHS Trusts in England) [21]
Develop a measure quantifying access to specialist TYA cancer care	Analysis of NHS HES Admitted Patient Care data
Workstream 2: Does specialist care make a difference	
Determine the outcomes associated with TYA cancer care	Longitudinal cohort study using a bespoke survey [23] administered 5 times over 3 years from 6 months after diagnosis
Determine carers experience of TYA cancer care	Unmet needs questionnaire administered to young people’s main carer nominated at wave 1
Workstream 3: How much does specialist care cost?	
Calculate the cost of TYA cancer care	Young person administered health economics questionnaires for out of pocket costs, analysis of clinical and HES data

*HES* Hospital Episode Statistics, *NHS* National Health Service, *TYA* teenage and young adult

### Establishing the BRIGHTLIGHT user group

Recruiting to the branding workshop had been possible with support and assistance from TYA clinical teams. This formed the foundation for establishing the BRIGHTLIGHT Young Advisory Panel (YAP). The initial YAP meeting had nine attendees from around England, predominately female (8:1) and aged 17–26 years (diagnosed with cancer when aged 14–25 years). Based on our previous experience, the YAP was established based on three key principles agreed between the research team and young people:

1. Need for Terms of Reference: centred on mutual respect, codes of conduct to ensure safety and reimbursement for time and travel.
2. Use of flexible communication methods: using preferred methods at the time such as Facebook, text, and email.
3. Support flexibility around commitment: acknowledging and accommodating changes in life situation, for example, finishing education and gaining employment. The invite to attend activities and offer advice is disseminated to all the YAP, members may not however engage for extended periods of time but they are only removed from the Facebook group at their request.

The YAP have met face-to-face in an annual workshop, otherwise communication has been through their Facebook page, email or telephone depending on level of engagement. The annual workshop is advertised to existing YAP members and members of the cohort who have consented to be contacted about BRIGHTLIGHT events. We also advertise on social media known to be used by young people.

### THE YAP and BRIGHTLIGHT set up

Young people were involved in the development and validation of the survey [23]. We held a YAP workshop in Leeds in August 2012 attended by four young people (one ex-CCG member, one consumer member of the NCRI TYA CSG and two people from a local TYA unit). They advised on publicity materials, newsletters and designed the website, which then underwent further modification in 2013. Their experiences of the workshop can be viewed here: https://jtvcancersupport.com/2014/04/brightlight-young-persons-advisory-panel/. At this meeting it was suggested we communicate through a closed Facebook page, unfortunately by the time the Facebook page was approved by local institutional regulations the young people had moved on or changed their email (as is common in this cohort) and only one responded to the invite to join the new Facebook group.

### The YAP and BRIGHTLIGHT project management

The YAP were also involved in aspects of study management that consumers are usually consulted on, such as designing the patient information sheet. Regrettably, we were unable to follow all of their recommendations, for example, to shorten them and use fewer words, because of the requirements of the research ethics committee at that time (2011). The information sheet for the BRIGHTLIGHT cohort study, a low risk survey, was nine pages long. However, we were later able to have a shorter version approved to be distributed with the longer version. Additionally, at the recommendation of the YAP we also created short audio videos of the information sheet, https://jtvcancersupport.com/2014/04/brightlight-patient-information/, a study description by the Chief Investigator, https://jtvcancersupport.com/2014/04/brightlight-jeremy-whelan/, and some introductions to team members who young people were contacted by during the study, https://jtvcancersupport.com/2014/04/brightlight-natasha-anita/.

The two key aspects of project management the YAP had the biggest influence were: 1) recruitment and 2) retention of BRIGHTLIGHT participants.

#### Recruitment interventions

BRIGHTLIGHT recruitment issues are well documented [11, 25]. The study aimed to recruit 2012 young people over 18 months, and recruited 1114 over 30 months, just over half the recruitment goal but an impressive figure for rare cancers in a rare population. We had a recruitment strategy in place at the onset of the study but with recruitment not being as we expected, we consulted our YAP. In 2013, the YAP annual workshop focussed on recruitment to research and methods of approaching young people about research, how this may vary by study type and to identify potential methods to improve recruitment. Eight of the YAP participated in a workshop to discuss aspects of recruitment to research including: whether it was appropriate not to tell young people about research; which members of the healthcare team were appropriate to tell them about different types of research; and what types of research it was appropriate to contact them by post or on social media [5]. Based on the YAPs advice we were able to implement a method of directly approaching young people to overcome potential gatekeeping as a barrier to recruitment [25]. We also trained additional treatment team members in recruitment.

#### Retention strategy

A key challenge in longitudinal research is retaining participants; maximising retention was particularly pertinent for BRIGHTLIGHT as recruitment was less than anticipated. We had considered the many life status changes and geographical mobility of young people and already had in place a mechanism for tracing young people. The study design accounted for participation in three out of the five data collection points (waves 1–5) to give young people the flexibility of ‘dipping in and out’ depending on their current life situation. Yet, by wave 3 (18 months after treatment) retention rates were a concerning 30%. In 2014, during their annual workshop, ten YAP members reviewed our existing retention strategy developed based on existing literature. Through a series of participatory activities additional actions were suggested, which we implemented. This included personalised letters prior to each wave of survey administration, including a postcard with key results, enhanced methods of tracing young people and a revamped website [15]. Implementation of these changes was associated with an increase in wave 3 participation from 30 to 58%.

## The YAP and dissemination

We wished to share results at as early a stage as possible with young people themselves. In 2014 two members of the YAP volunteered to attend FYSOT to present demographics and emerging findings from the BRIGHTLIGHT study. Two YAP members developed the slides supported by Lorna Fern and delivered the presentation and took questions from the audience (https://jtvcancersupport.com/2014/11/fysot-14-brightlight/). BRIGHTLIGHT results were shared with 350 young people at FYSOT and the live streaming provided by JTV Cancer Support.

With other results emerging from various streams in the BRIGHTLIGHT programme of work, we sought ways to disseminate meaningfully to a wider, lay audience. Additional funding was obtained allowing us to fully immerse BRIGHTLIGHT in the world of theatre. We worked with Brian Lobel, a Wellcome Trust Public Engagement Fellow and Contact Young Company, a young theatre group (https://contactmcr.com/project/contact-young-company/). Collaboratively the research team, YAP members and TYA nurses worked with the theatre company to offer an interpretation of our results so far. The cast were joined by four young people with cancer to facilitate that interpretation and add context. What resulted was a script for a theatrical performance, named ‘*There is a Light: BRIGHTLIGHT’*.

*There is a Light: BRIGHTLIGHT* took eight weeks to develop from the first workshop to the opening night at the S!CK Festival (www.sickfestival.com) in Manchester in March 2017. It played for three nights then toured to public audiences in Brighton, London and Edinburgh, to delegates of the Royal College of Nursing International Research Conference in Oxford and delegates at the NCRI Annual Conference in Liverpool and the final performance was to young people attending FYSOT. ‘There is a Light’ was viewed live by over 1200 people, the live streaming on one night has been viewed more than 650 times and a documentary accompanying the performance is now publicly available https://vimeo.com/238045094. The impact evaluation is underway but comments from the audience suggest this was a meaningful way of conveying results from research to both professionals, patients and the public:

*“I have worked with children and young people with cancer for nearly 30 years and read many dry research reports. This was exciting, meaningful, understandable, I'm sure I will remember the detail for much longer than the hundreds of papers I've read before and I will talk about it. Thank you”*

*“Found it really hard hitting and impactful and made me consider several areas of my life post diagnosis that I still haven't 'dealt with' and are not that different even when older.”*

*“For a play about young people with cancer, unexpectedly life-affirming, warm, vital & real. From strength of responses in post-show, seemed there is a lack of platforms for researchers/medical experts, people affected by cancer to share experiences - thanks for going some way to changing that.”*

We also worked with the YAP on relaying results to healthcare professionals and academic audiences. The YAP had an integral role in a joint education meeting with TYAC (the professional organisation for healthcare professionals working with young people with cancer in the UK). Two YAP members acted as co-chairs, proving themselves very effective at drawing the audience into interactive discussion. Their feedback about participating in a scientific conference was positive: “*I really enjoyed myself, found it so informative and it was awesome to see this aspect of it all*”.

## The YAP and generating future evidence

We invited the YAP to offer ideas and suggestions for issues where they felt there was a lack of evidence or where care needed to change. Two new areas for study emerged.

### SEX, body image and relationships

As a consequence of discussion at the 2014 annual workshop the YAP requested a workshop focusing on sex, body image and relationships. We restricted this workshop to YAP members over 16 years in respect of factors including the intimate subject matter, the need for young people to feel safe and the UK age of consent for sexual activity. The workshop was led by an external facilitator, with a previous cancer diagnosis as a young adult and was expert in communicating about sex, body image and relationships. Five YAP members attended and through a series of creative activities and group discussion they identified the following overarching themes (i) information sharing; (ii) contexts and relationships (influencing factors); and (iii) information sharing preferences [16]. The depth of discussion within this workshop warranted a second workshop where we had hoped to invite partners; however, while a number of new YAP members attended the second workshop we have not yet managed to reach partners. The evidence from these workshops is being used to support a grant application with the YAP as co-applicants to address communication about sex by healthcare professionals when young people are diagnosed with cancer.

### Generating hypotheses for secondary data analysis

**Fig. 3:**
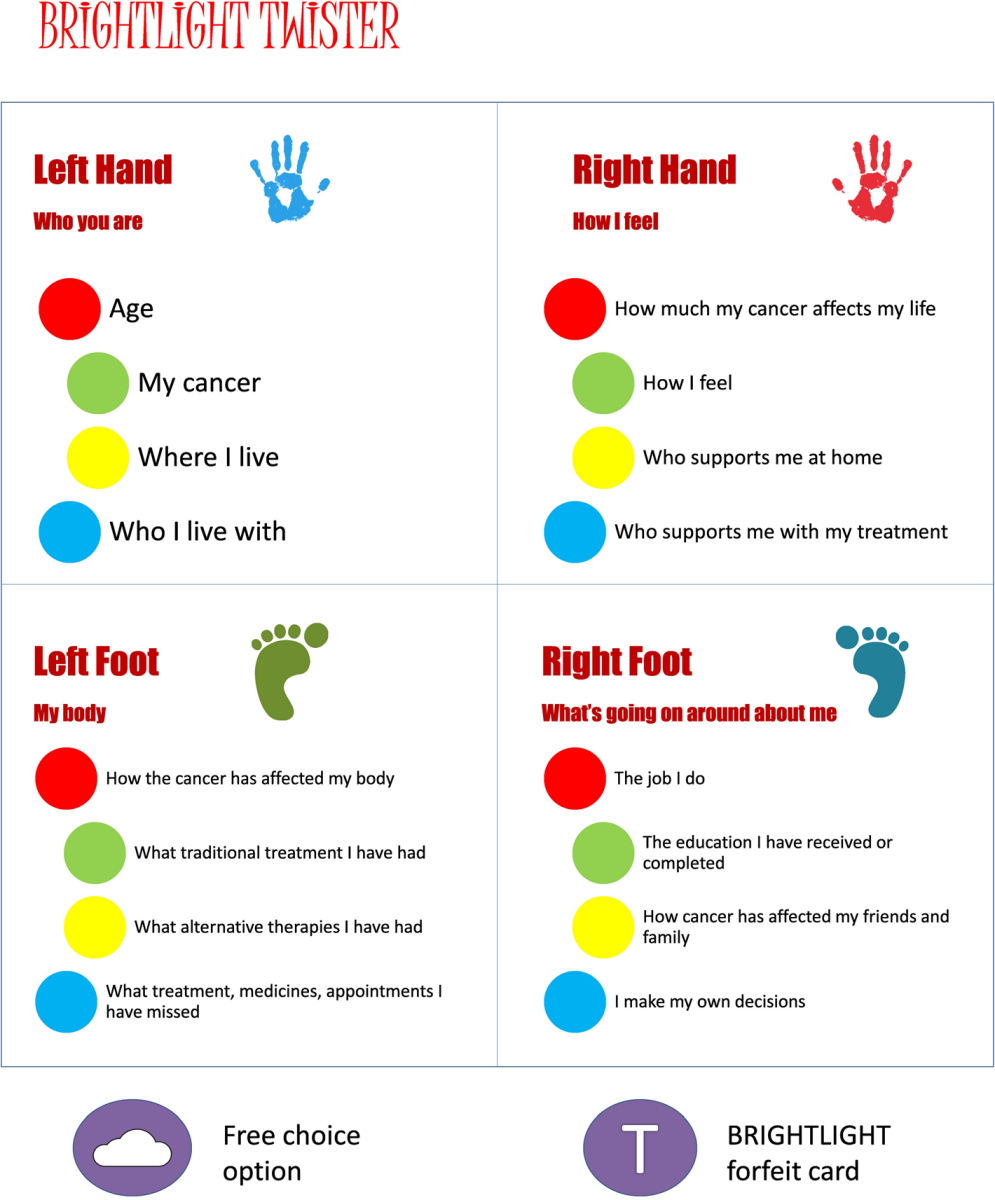
Links between *Twister*™ moves and aspects of BRIGHTLIGHT data

We sought the YAPs views on topics for secondary data analysis of the BRIGHTLIGHT dataset. Eight members of the YAP attended a one-day workshop and were divided into two teams (BRIGHT & LIGHT); an adaptation of the board game *Twister™* was used to create links between different aspects of data (Fig. 3). Two members from each team were on the mat at any one time; the other members spun the wheel. Teams made alternate spins until all four players had made a move, the links between each team’s moves were recorded then each team discussed how they thought the two domains were linked.

Thirty-six links were discussed, generating 21 hypotheses or research questions. These were circulated to the whole of the YAP to vote for their top three (Table 5). The results of the recent James Lind Alliance research prioritisation exercise [26] will allow us to ascertain which of the 21 questions should be prioritised.

**Table 5:** The top three research questions generated through BRIGHTLIGHT *Twister*™

1. Is the impact of cancer affected by how much support you get from people in similar situations? 2. How I feel about my body after a cancer diagnosis affects my ability to form new relationships? 3. =Am I less likely to be involved in decision-making if I am younger? 3. =How much information I received from my treatment team affects how I feel about myself when treatment finishes?

**Table 6:** Guidance for researchers: the Seven Ps of PPIE

1. Passionate People	• The right professional team is crucial. If the team do not believe in PPIE then it will be difficult to move beyond tokenism. • Always have a key person for people to contact, who will be the main link into the team. Decide in advance who this will be and allow sufficient resource for that person to manage the group. This person has to be committed and passionate about PPIE
2. Preparation	• Take time to think about what you want your group to do. Be clear with them about their tasks and input. • Be open to different ideas if planned activities are not viewed favourably by the group. • Good PPIE events take time to plan and execute. Allow sufficient time to make documents explaining complex ideas. Decide in advance who is going to do this. • Ensure you have the aims of face-to-face workshops clearly visible during the meeting. • Let participants know in advance what they will be working on so they have time to think about their experience and what they can contribute. • Consider how people are going to travel to face-to-face meetings. Who is responsible for sorting out travel tickets, room bookings, and reimbursement? • Is the venue too clinical and so may inhibit free speech about their experiences? • Is the venue accessible to those with disabilities. • Have an agenda with flexible break times, the content of the workshop may mean breaks are needed sooner or later than initially thought, you cannot always predict when the group or the researcher will need to break. • If the group are presenting, who is preparing the slides? Who is going to practice with the group prior to presentation? • Plan for a member of the team to contact each PPIE member individually after the event to ensure no distress. • Have in place processes for sickness, adverse weather, travel disruption and a key contact for participants to contact by phone. • Prepare an evaluation sheet and seek feedback.
3. Practice	• Events will get easier and better with time: the more you do the more familiar you will become. • Just like professional groups, PPIE groups can take a while to gain momentum. • Take notes after each event of what went well and what did not, then build on processes or activities that worked with that particular group. • Make a list of venues participants particularly like or dislike. • Use the evaluation feedback to build on previous events.
4. Pounds	• PPIE can be resource intensive, plan a budget in your grant application. • At submission stages if you have no budget to reimburse time, explain to participants prior to the event, or utilise existing subsidised groups. • Reimburse travel and provide food as a minimum. • If participants are leaving at lunch time or dinner time provide food or food vouchers such as supermarket or restaurants. • When costing a grant, budget for relaxed creative environments, workshop materials such as printing and props, travel and overnight stay if you think needed and conference costs for participants to present. • Budget for open access publication of your PPIE.
5. Perseverance	• It may take a while to find the right people to become involved. The group may be in flux establishing its core members and this can take time.
6. Post it notes	• Post it note methodologies are useful, however there are a realm of activities in healthcare and beyond, which lend themselves well to PPIE. • Look outside of your normal genre for suitable activities, for example advertising, business trouble shooting activities. • Always have post it notes and glitter in your tool box for PPIE.
7. Patience	• Effective PPIE groups and events can take time to establish. • Complex research ideas can take time to explain and write down. • Be patient with yourself, your group and colleagues as you all learn how to work together.

## Overall reflections and discussion

**Fig. 4:**
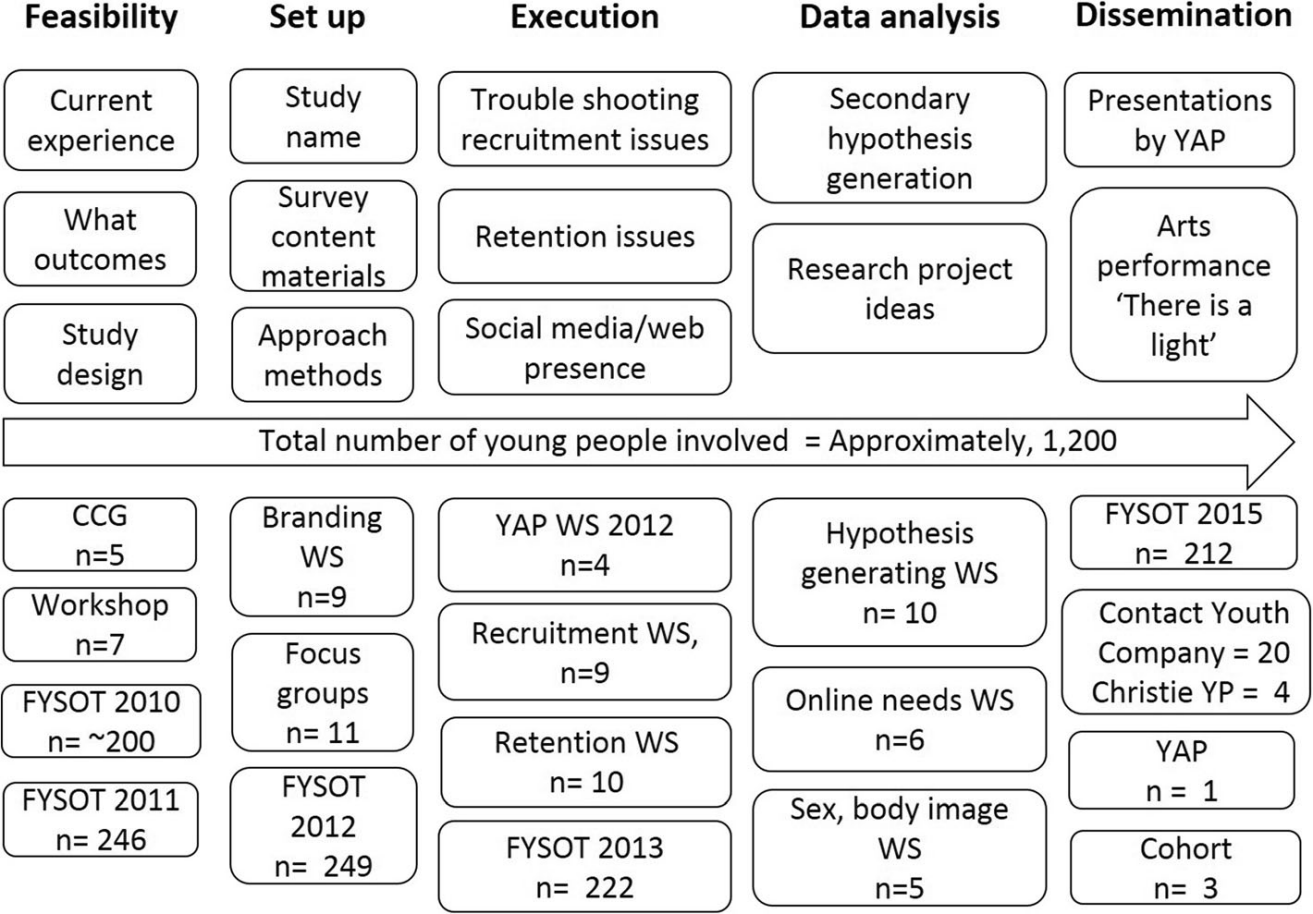
Summary of how young people have informed BRIGHTLIGHT

We have presented our reflections on 10 years’ experience of involving young people in research. We cannot say how different the final BRIGHTLIGHT study might have been if all the preceding steps had not been in place, and the CCG and the YAP had not informed all stages of development work. To the research team, their input has felt rich and indeed invaluable, especially in identifying solutions to the many challenges we have faced in this decade of work. Many of these we would not have considered and all their suggested changes were accepted as amendments unconditionally by the Research Ethics Committee. The CCG and YAP are valuable members of our team and through them we have managed to involve through consultation and collaboration over 1200 young people, approximately the same number of participants in the BRIGHTLIGHT study (Fig. 4).

Young people with cancer (and other long-term conditions) are often described as vulnerable [27, 28] and this ‘label’ often excludes them from opportunities to participate in healthcare research. For healthcare professionals and researchers with expertise in working with young people we believe with appropriate planning and support they can be empowered to actively contribute to the research agenda. With this in mind, we also ensure that involvement in activities requiring the YAPs time is enjoyable as well as productive and critically is also something they will get some benefit from in the future, for example confidence in public speaking. We also make sure they know that their contribution has made a difference, so feedback and managing expectations has been key to their sustained involvement. Each workshop was accompanied by an evaluation form and completed by each YAP member.

One criticism of our work may be the lack of formal evaluation of the effectiveness of young peoples’ involvement. Although there is now an increasing body of literature available addressing the evaluation of PPIE [7] this is quite recent and was not available at the start of our study. Additionally, the resources required to set up BRIGHTLIGHT were greater than anticipated and coupled with recruitment problems meant time to design and implement formal evaluation of PPIE was unavailable to the team. Through our reflections we have been able to present a narrative of evidence of the value of including young people as a central component of our research team. For example, the impact of the YAP retention interventions doubled our retention rates [15].

### Benefits for the YAP

It has been reported previously that young people participate in research for altruistic reasons [29]. This may be a motivation for young people joining the YAP but we believe that our processes and management of PPIE contribute to their continued involvement. Many of the YAP/CCG have gained skills they may have missed out on during treatment and thus can demonstrate on their CV or portfolio where they have ‘made use’ of their time out of education/work. Skills include: public speaking; being part of a group; facilitating focus groups; undertaking research interviews; problem solving; influencing study design, data collection, and analysing data; discussing complex ideas and making them simple; and interacting with a wide range of young people and healthcare professionals. There are also additional, practical/social skills such as travelling alone, spending time away from home after treatment and attending professional social engagements, that we might suggest help build confidence. For some young people attending YAP events this was the first time they had travelled alone and/or stayed away from home alone, since being diagnosed.

Young people have continued to be engaged in the YAP and our workshops have good representation, which we think reflects the esteem the research team affords them and the value we assign to their contribution. Additionally, we have some responsibility for supporting the YAP in their future endeavours. It is made clear to young people when they join the YAP that we do not replace any role undertaken by their healthcare teams but we are able to direct them to support, in the main we provide certificates of participation and supporting letters to include in their portfolios when leaving school/college/university as evidence of engagement during their treatment. For those interested in careers in healthcare we are able to signpost them to appropriate academics to provide career guidance.

We think one of the key benefits of being in the YAP is giving young people the space to realise a cancer diagnosis does not stop them from continuing their lives; contact with other members of the YAP and the professional cancer community gives them the confidence to find their ‘new normal’. Members of the CCG and YAP have gone on to contribute to other cancer-related activities, such as being Trustees of national charities and membership of other CSGs. To quote Amy, after participating in *There is a Light: BRIGHTLIGHT*

*“Before this I had a spine operation so I literally couldn’t walk for a while. Doing this got me out of the house, out of my comfort zone really so it’s been good for me. Confidence-wise, I’ve gone from zero to all the way up here!”*

### Challenges of PPIE with young people

We present the benefits of involving young people in research but we also need to acknowledge that there are a number of challenges; some of these have been reported previously [2–4] but some of these may reflect the uniqueness of this age group.

#### Evolving technology

Young people are immersed in technology [30], keeping up with this as researchers working in an NHS environment is challenging and brings with it additional safeguarding issues. Young people asked for the main mechanism for communication to be through Facebook as young people do not regularly use email (https://techcrunch.com/2016/03/24/email-is-dying-among-mobiles-youngest-users/); however, access to Facebook was not allowed on NHS computers so a lengthy approval process was required to get access. By the time approval was granted the young people who initially requested this had moved on and we lost three potential YAP members. We started with Facebook which was quickly superseded by suggestions for a Twitter account, followed by Instagram and then Snapchat. As researchers, we had to consider the value of an online presence versus the resources required to manage the more recent social media channels which require more frequent engagement, such as snapchat. We have maintained a closed Facebook page and a public facing Twitter account.

#### Life stage commitments and workshops

Identifying agreeable times for the workshops has never been satisfactory as one group will always be excluded; if it is organised around college/university term times those in work are often unable to attend and if organised at weekends, this excludes those with families and most often those at University have weekend employment. We therefore vary when we hold workshops to be as inclusive as possible. We have also varied workshop location around the country in a bid to bolster numbers, although approximately 10 young people per workshop is an impressive number. We have not found an ideal solution to times and location for workshops and feedback from young people have confirmed that the weather will also influence attendance, either “too cold to go out” or “too warm to be inside”.

#### Cost

One of the biggest challenges with running the YAP is they are a national group so there is a substantial cost incurred whenever we bring them together face-to-face. Even with young people’s rail cards, there is a significant cost of train travel and if a young person lives in a part of the country where they are unable to get to London in time for the workshop, we will provide hotel accommodation. Furthermore, some of the CCG/YAP have disabilities associated with their cancer and some were too young to travel alone and needed help from an accompanying person, which we also provide for. We have the benefit of NIHR funding to support this activity but for fledgling projects with limited funding there may not be the opportunity to optimise PPIE. Having said this, the value of face-to-face interactions for the group should not be underestimated. It is essential in maintaining momentum and contribution to the overall study. Researchers should fully cost PPIE activities and include realistic amounts of funding when submitting grant applications to ensure patient’s involvement is optimised throughout the duration of the study.

#### Moving on

It is important to facilitate transitioning young people out of user involvement in order to allow them to fully integrate back to their previous lives and to allow new opportunities for those with a more recent diagnosis. This generally happens naturally as young people move further from their diagnosis and their lives become populated with going back to education or work. For those who want to remain involved in research in a professional capacity we offer a more structured role of co-researcher with subsequent studies which are of relevance.

#### Representation of the population

Our YAP are a self-selected group of young people, who are well informed about research, cancer and are familiar with PPIE. As such they may not represent the views of all young people and in fact, they have become experts in teenage and young adult cancer research. For this reason, we consult participants attending FYSOT to confirm the YAPs views.

## Conclusions

Policy and practice encourages PPIE, PPIE is at the core of the work of the NHS, some funding bodies ‘mandate’ it, and some funding bodies have their own PPI groups, which researchers can access when first designing new studies. While there are challenges to involving young people in healthcare research, we hope we have evidenced to the reader that the benefits outweigh these, and that PPIE is a crucial endeavour in all our research. Furthermore, for populations who are defined as ‘vulnerable’ or ‘hard to research’, a ‘label’ given by the professional community, we hope we have also shown that taking the time to involve these populations produces results; sometimes this just requires a little more creativity and support. This is an evolving area of research practice, but the more we share, the more we learn from each other. We will always need to challenge systems, where there is a ‘poor fit’ with our PPIE plans. For example, we have not been able to realise one of our early decisions, to include young people as co-authors in published manuscripts. We included them in one of the early Essence of Care publications [13] but the journal would not waive the need for co-author approval in their standard format (we could provide copies of our consent forms). The steps required to set up electronic signatures resulted in unnecessary burden for the CCG so with young people’s agreement we now include the YAP in the acknowledgements rather than as co-authors. Systems and processes will have to catch up, in the same way that we have to keep up with social media to maximise youth involvement. Finally, we would like to conclude by offering guidance to other researchers who are involving consumers in their projects through what we title ‘the 7Ps of PPIE’ (Table 6).

## Abbreviations

CCG: Core Consumer Group
CSG: Clinical Studies Group
FYSOT: ‘Find Your Sense of Tumour’
JLA: James Lind Alliance
NCRI: National Cancer Research Institute
NICE: National Institute for Health and Care Excellence
NIHR: National Institute for Health Research
PPIE: Patient and Public Involvement and Engagement
TYA: Teenage and Young Adult
UK: United Kingdom
YAP: Young Advisory Group

## Footnotes

1 The core consumer group attended a nationally recognised patient and public involvement training event specifically for patients with cancer, funded and ran by a national cancer charity. In addition researchers Faith Gibson, Rachel Taylor and Lorna Fern provided training and support prior to each workshop.
